# Statistical learning of spatiotemporal regularities dynamically guides visual attention across space

**DOI:** 10.3758/s13414-022-02573-5

**Published:** 2022-10-07

**Authors:** Zhenzhen Xu, Jan Theeuwes, Sander A. Los

**Affiliations:** 1 Department of Experimental and Applied Psychology, Vrije Universiteit Amsterdam, Van der Boechorststraat 7, 1081 BT Amsterdam, The Netherlands; 2 Institute Brain and Behavior Amsterdam (iBBA), Amsterdam, The Netherlands

**Keywords:** Visual attention, Spatial attention, Temporal attention, Spatiotemporal regularities, Statistical learning

## Abstract

In dynamic environments, statistical learning of spatial and temporal regularities guides visual attention in space and time. In the current study, we explored whether and how combined spatiotemporal regularities regarding target events guide visual attention. In three experiments, participants performed the additional singleton task. They were asked to search for a target stimulus with a unique shape among five non-target distractors and respond to the orientation of a line inside the target. Unbeknownst to the participants, the moment in time that the search display was presented was predictive of the target location. Specifically, the target was more likely to be presented at one high-probability location after a short interval and at another high-probability location after a long interval. The results showed that participants’ performance was better for high-probability locations than for low-probability locations. Moreover, visual search efficiency was greater when the target appeared at the high-probability location after its associated interval than when it occurred there after its nonassociated interval, regardless of whether the distribution of intervals was uniform (Experiment 1), exponential (Experiment 2), or anti-exponential (Experiment 3). Taken together, the results indicate that implicitly learned spatiotemporal regularities dynamically guide visual attention towards the probable target location.

**Supplementary Information:**

The online version contains supplementary material available at 10.3758/s13414-022-02573-5.

In a dynamically changing world, it can be quite challenging to extract information that is relevant for the ongoing task. To cope with this problem, it has been shown that individuals implicitly use spatial and temporal regularities that the environment provides (Fiser & Aslin, 2001; Saffran et al., 1996). In particular, it has been revealed that statistical learning (SL) of spatial and temporal distributions of events biases visual attention to relevant locations and relevant time points (Olson & Chun, 2001; Turk-Browne et al., 2005; Wagener & Hoffmann, 2010; Wang & Theeuwes, 2018a), which is consistent with the selection history component in the taxonomy of attentional control proposed by Awh et al. (2012; see also Failing & Theeuwes, 2018; Theeuwes, 2019).

The idea that SL of spatial regularities biases visual attention is not new. In early studies of contextual cueing, it was found that participants are able to implicitly learn the associations between spatial configurations and target locations. Target localization and discrimination are facilitated when targets consistently appear at a specific location within an earlier presented context than when they appear at a location within a new context even if participants have no explicit knowledge about the contexts (Chun & Jiang, 1998). This benefit suggests that the visual context serves as a cue which implicitly guides spatial attention to the potential target location (Chun & Jiang, 1999). In addition, it has also been shown that visual search efficiency for targets is higher when the target appears at probable locations than when it appears at improbable locations while participants are unaware of the distribution regularities (Geng & Behrmann, 2002, 2005; Jiang et al., 2013), suggesting that spatial attention is oriented implicitly towards probable target locations. Similarly, visual search efficiency for targets has been found to be higher when distractors appear at high-probability distractor locations than when they appear at low-probability distractor locations, indicating that SL of distractor regularities guides spatial attention away from probable distractor locations (Wang et al., 2019; Wang & Theeuwes, 2018a, b, c).

Alongside the study of attentional orienting to locations in space, there has been growing interest in the study of attentional orienting to moments in time (Coull & Nobre, 1998; Miniussi et al., 1999; Nobre & van Ede, 2018). Within this field, it has been shown that temporal regularities of target presentation may come to expression in performance measures, suggesting that, due to SL of temporal regularities, moments in which the target presentation is expected are prioritized over other moments in time. In an early study, Olson and Chun (2001) manipulated the sequential structure of visual events, in which the timing of the next element could be predicted from the temporal position within a sequence. They found that this sequential temporal context could be implicitly learned and come to guide visual attention to a point in time. Subsequent studies confirmed that SL of temporal order regularities biases visual attention in an automatic and implicit way (Li & Theeuwes, 2020; Turk-Browne et al., 2005; Wang et al., 2021; Zhao et al., 2013).

To study how attention comes to prioritize certain time points over others, researchers have varied the foreperiod, which refers to the interval between a neutral cue and the target stimulus (Niemi & Näätänen, 1981). During this interval, a postulated process of temporal preparation or expectation develops, which facilitates responses to the target stimulus (e.g., Los et al., 2014; Salet et al., 2022). The time course of general temporal preparation has been shown to be tuned by the context of intervals. When the interval randomly varies from trial to trial according to a uniform distribution, participants respond faster and more accurately to the target stimulus as the interval lengthens. When the proportion of short intervals increases, the function relating reaction time (RT) to interval gradually lies down, and it becomes approximately flat in the case of an exponential interval distribution. By contrast, when the proportion of long intervals increases, the RT-interval function gradually becomes steeper (Los et al., 2017; Los et al., 2021; Niemi & Näätänen, 1981; Trillenberg et al., 2000; Vangkilde et al., 2013). There is ongoing controversy as to whether general temporal preparation is driven by the hazard function (Nobre et al., 2007; Nobre & van Ede, 2018; Visalli et al., 2019; Visalli et al., 2021) or by learned memory representations of time (Los et al., 2017; Los et al., 2021; Salet et al., 2022). It is furthermore unclear which process(es) are facilitated by temporal preparation. Some studies favor a motor or premotor locus for the effect of temporal preparation, while others claim that temporal preparation affects spatial attention (Rolke & Ulrich, 2010; Vangkilde et al., 2013). In addition, the role of arousal and alertness are also mentioned in relation to temporal preparation (Hackley & Valle-Inclán, 1998; Steinborn & Langner, 2012). Regardless of how temporal preparation is explained, there is consensus that it reflects a gradual learning of the possible moments in time for either target occurrence or response execution.

Recently, researchers have started to combine paradigms of spatial and temporal learning. This work has shown that task performance is influenced by SL of spatiotemporal regularities when the interval provides information about the location or identity of upcoming events (Thomaschke & Dreisbach, 2013; Thomaschke et al., 2011). For instance, Wagener and Hoffmann (2010; Experiment 2) used a choice RT task, in which a target stimulus was presented at one of two possible locations after one of two possible intervals. Critically, the interval indicated the location at which the target was most likely to appear. The target was presented more frequently at one location after a short interval and at another location after a long interval. They found that participants responded faster and more accurately when the target appeared at a temporally valid location than appeared at a temporally invalid location, revealing the behavioral expression of spatiotemporal regularities about target events. However, on the basis of this study, it is difficult to determine what caused this behavioral benefit. On one hand, it could reflect visual attentional orienting, resulting in faster processing of a stimulus that appeared at the likely location. On the other hand, it could reflect motor preparation, resulting in a faster motor response to a stimulus appearing at the likely location (Thomaschke et al., 2011; Wagener & Hoffmann, 2010).

To disentangle these two accounts, Thomaschke et al. (2011) used four target stimuli which differed along the dimension of orientation (vertical, horizontal) and shape (diamond, oval) in a speeded binary choice task. One of these dimensions determined the response, while the other dimension was response-irrelevant. In addition, one of the dimensions was correlated with the preceding interval, while the other dimension was not. The critical finding was that time-event regularities only affected performance when the response-relevant dimension was correlated with the interval, not when the response-irrelevant dimension was correlated with the interval. Thus, they concluded that the effect of the time-event regularities was response specific. Subsequent studies confirmed that motor responses play an important role in the learning of time-based event regularities (Thomaschke & Dreisbach, 2013; Volberg & Thomaschke, 2017).

At the same time, other studies provided evidence in favor of the attention account. In a Posner spatial cueing task, Rieth and Huber (2013) implicitly manipulated the validity of the exogenous cue based on the interval between the cue and the target stimulus. For one group, the target appeared on the cued side after a short interval and on the uncued side after a long interval, while for another group this contingency was reversed. As participants’ task was to press a key after the appearance of the asterisk target stimulus, the response was independent of the spatiotemporal regularities of the target. The two groups showed differential attention orienting effects. The cueing effect was significantly larger for the short invalid/long valid group, while inhibition of return (IOR; Klein, 2000) was significantly larger for the short valid/long invalid group. These findings remained in place during a test phase in which the regularities were removed, indicating that the interval-based spatiotemporal regularities about the targets were implicitly learned. Based on these findings, they argued that spatial attention implicitly adapts to spatiotemporal regularities.

In a recent eye-movement study, Pfeuffer et al. (2020) associated two intervals with two target locations. The interval predicted the upcoming target location with 100% validity. The results showed that participants first moved their eyes to the location associated with the short interval. If there was no stimulus presented after the short interval had passed (trials with the long interval), then they looked towards the location associated with the long interval. While revealing the adaptation of overt attention to implicit spatiotemporal target regularities, this design is somewhat impoverished because only one stimulus appears at either one of two locations.

Boettcher et al. (2022) explored the adaption to the spatiotemporal regularities of a target in a noisy visual search task. Participants were asked to search for eight instances of a target (vertical line) that faded in and out of a display containing similar transient distractors (tilted lines). If the target was found, they had to click on it. On each trial, the presentation of four of the eight targets was spatiotemporally predictable, as each target appeared in a specific quadrant at a specific time point within the trial. The other four targets appeared randomly in time and space within each trial. Participants’ performance was significantly better for spatiotemporally predictable targets than for unpredictable targets. The corresponding eye-movement data also showed a higher probability to fixate the target quadrant for predictable compared with unpredictable targets. Based on these findings, the authors concluded that spatiotemporal regularities guide attention in dynamic visual search. This study is important because it extends the findings of SL of spatiotemporal regularities to noisy dynamic contexts. However, the findings do not necessarily imply that participants have learned to orient attention to a particular location and moment in time. Instead, participants may have learned that they needed to make a motor response towards a particular quadrant at certain time points. Also, since the detection of the blurry target among noisy distractors required eye movements, participants may have learned that they needed to make a saccadic eye movement to detect the target that was presented at particular quadrants at particular moments in time. Therefore, this study does not necessarily show learning of spatiotemporal attentional orienting, as it could reflect spatiotemporal (saccadic) motor learning instead.

As time-based motor preparation plays an important role in explaining the behavioral expression of the spatiotemporal regularities, studies in which the motor response is independent of the spatiotemporal regularities would provide more convincing evidence for the notion that attentional orienting can be guided by spatiotemporal regularities. A recent study of Xu et al. (2021) did exactly this by using the additional singleton paradigm (Theeuwes, 1991, 1992) to explore the effect of spatiotemporal regularities regarding the salient distractor. Participants searched for a unique target shape (e.g., a diamond) presented among five differently shaped nontargets (e.g., circles) and responded to the orientation of a line within the target. On the majority of the trials, one of the nontargets had a unique color (the color singleton distractor). Critically, the distractor appeared relatively frequently at one high-probability distractor location after a short interval and at another high-probability distractor location after the long interval. The results showed higher search efficiency for targets when the distractor appeared at a high-probability location after its associated interval than when it appeared at that location after the other interval. Since the motor response was fully separated from the spatiotemporal regularities in this study, these findings indicated that attention was dynamically guided away from the probable distractor location at the specific moments in time through SL of the spatiotemporal regularities.

The current study investigated whether spatiotemporal regularities of target events dynamically guide attention towards the probable target location. To that end, we adopted the paradigm used in Xu et al. (2021) but without a colored singleton distractor. Participants were instructed to search for a unique shape singleton presented among five nontargets and they responded to the orientation of the line inside it. To minimize eye movement learning, participants were asked to keep their gaze at the center of the screen all the time during this covert attention task. Following the fixation dot, the search display was presented either after a short or long interval. Unbeknownst to participants, the target appeared more frequently at one high-probability target location after a short interval and at another high-probability target location after a long interval. The probability that the target appeared at any one of the other four low-probability locations after either interval was equal. If spatiotemporal regularities dynamically guide attentional selection, performance should be better if the target is presented at the temporally congruent high-probability location than when it is presented at either the temporally incongruent high-probability location or at any one of the low-probability locations. We tested these predictions across three experiments, in which we used different distributions of intervals (uniform, exponential and anti-exponential distribution) to ensure that the spatiotemporal guidance of attention was independent of general temporal preparation.

## Experiment 1

In Experiment 1, we tested whether participants could learn spatiotemporal regularities regarding the target with two equally likely intervals (i.e., under a uniform distribution, 1:1). From the onset of the fixation dot, the search display was presented with equal probability after either a 500-ms interval or a 1,500-ms interval. Critically, the target appeared more frequently at one high-probability target location after a short interval, while it appeared more frequently at the opposite high-probability target location after a long interval. We expected that attentional selection would be dynamically guided by spatiotemporal regularities, so the temporally congruent high-probability target location should facilitate search relative to either the temporally incongruent high-probability location or any one of the neutral locations.

### Method

#### Participants

The sample size was pre-determined based on the effect size observed by Wagener and Hoffmann (2010). To observe the reported effect of η_p_^2^ = .35 with .80 probability (α = .05) in a 2 × 2 (interval × target location) repeated-measures analysis of variance (RM-ANOVA), MorePower 6.0.4 (Campbell & Thompson, 2012) suggests a sample of 18 participants. However, we aimed for a much larger sample size of 40 participants because the experiment was conducted online. To this end, we measured 47 participants, of whom six were excluded because of low accuracy (less than 70%). Participants were compensated by credits or payments. The final sample thus consisted of 41 participants (age 20.0 ± 3.37 years, 30 females), including 31 participants recruited from the Vrije Universiteit Amsterdam and 10 participants recruited from Prolific. Participants all gave written informed consent at the beginning of the experiment, and this study was approved by the Ethical Review Committee of the Faculty of Behavioral and Movement Sciences of the Vrije Universiteit Amsterdam.

#### Apparatus

The experiment was built and run on OpenSesame/OSWeb (Version 3.3.9b; Mathôt et al., 2012). The programmed experiment was hosted by JATOS. Participants were directed to the experiment on JATOS by the participant recruitment platforms Prolific and SONA (VU). Participants were instructed to perform the experiment in a quiet environment on a laptop or PC, and to turn off all possible sources of distraction.

#### Procedure and design

**Fig. 1:**
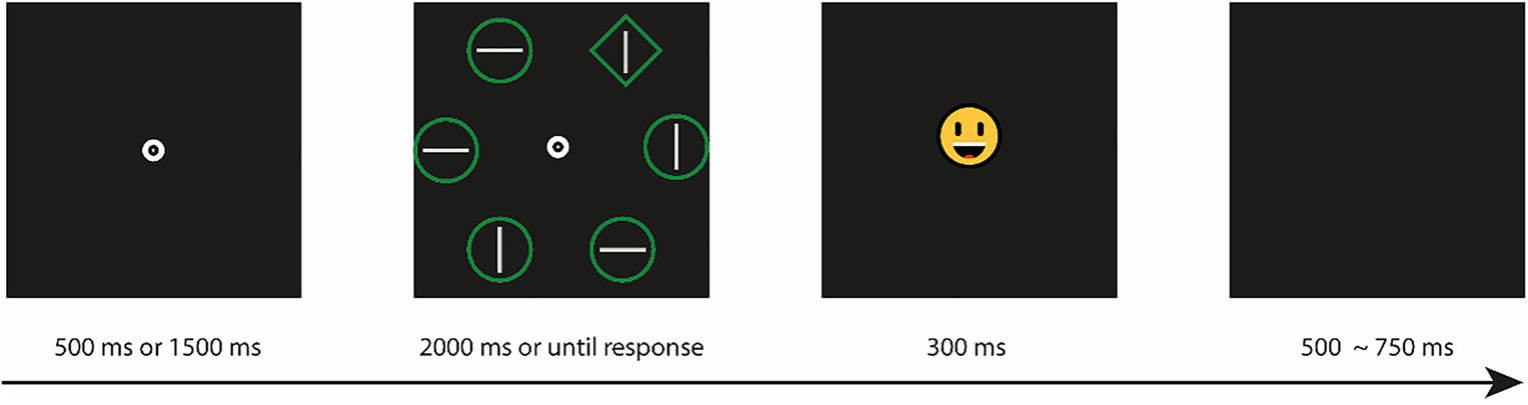
The sequence of trial events in Experiments 1, 2, and 3. *Note*. Each trial started with the presentation of a white central fixation dot for either 500 ms or 1,500 ms. Then the search display was presented for 2,000 ms or until a response was made. Participants were asked to search for the unique shape (the diamond in this example) among five nontargets (i.e., the circles) and respond to the orientation of the line inside it. The target could appear at any one of the six locations but had a higher probability of occurring at one location (the “high-short” location) after the 500-ms interval and at the opposite location (the “high-long” location) after the 1,500-ms interval. As soon as a response was made or the search display timed out, a feedback display was presented for 300 ms. The intertrial interval jittered from 500 ms to 750 ms. Stimuli are not drawn to scale. (Color figure online)

As shown in Fig. 1, each trial started with a central white fixation dot against a black background. The fixation dot was a white filled circle (8-pixel radius) with a central hole (2-pixel radius). After an interval of either 500 ms or 1,500 ms, a search display consisting of six shapes arranged on an imaginary circle (210-pixel radius) around the central fixation dot was presented for 2,000 ms or until a response was made. These shapes were either a diamond (112 × 112 pixel) among five circles (51-pixel radius) or vice versa. All shapes had either a red or green outline (randomly varied across trials) with a gray line oriented horizontally or vertically inside it. Participants were asked to search for the unique shape and respond to the orientation of the line inside it by pressing the “z” or “m” key. They were instructed to fixate at the central fixation dot throughout a block of trials and to respond as quickly and accurately as possible. After the response or search display timeout, a feedback display (57 × 57 pixel) with either a happy face (correct response) or a sad face (incorrect response) was presented for 300 ms. The intertrial interval was from 500 ms to 750 ms.

For each participant, two opposite locations were designated as high-probability target locations and denoted as the “high-short” and “high-long” target locations. The three possible pairs of high-probability target locations as well as the location assigned to the high-short and the high-long condition within each pair were randomized across participants. In half of the trials, the interval between the onset of the fixation dot and the search display was 500 ms. Across all trials with the 500-ms interval, the target was more frequently presented at the high-short target location (64.3%) than at either the high-long target location or any of the four low-probability target locations (7.1% each). In the other half of the trials, the interval was 1,500 ms. Across all trials with the 1,500-ms interval, the target was more frequently presented at the high-long location (64.3%) than at either the high-short location or any of the low-probability target locations (7.1% each). All conditions were randomized in each experimental block (shown in Table 1). The experiment consisted of a practice block of 25 trials and 6 experimental blocks of 112 trials each.

**Table 1:** Number of trials in each condition of each experimental block in Experiments 1, 2, and 3

Target location	High-short	High-long	Low	Total
Interval (ms)				
Experiment 1				
500	36	4	16	56
1,500	4	36	16	56
Total	40	40	32	112
Experiment 2				
500	36	4	16	56
1,500	2	18	8	28
Total	38	22	24	84
Experiment 3				
500	18	2	8	28
1,500	4	36	16	56
Total	22	38	24	84

*Note.* There were four low-probability target locations, so the probability that the target appeared at any one of these locations was equal to the probability that the target appeared at a high-probability target location after its nonassociated interval.

At the end of the experiment, we tested participants’ awareness about the regularities. First, participants were asked to indicate at which two locations they thought the target appeared most often to estimate their awareness about the spatial distribution of targets. Then, participants were asked to indicate the frequent target location after a short interval and the frequent target location after a long interval to estimate their awareness about the spatiotemporal regularities.

#### Data analysis

Trials with RT below 200 ms or above 2,000 ms (1.68% of all trials) were excluded. For the analysis of RT, incorrect trials (10.79 % of all trials) and trials with RTs outside ± 2.5 standard deviations of the condition mean for each participant (2.27 % of all trials) were also excluded.

We analyzed the filtered RT data with linear mixed models (LMMs) and the accuracy (ACC) data with generalized linear mixed models (GLMMs) using the *lme4* package (Version 1.1.29; Bates et al., 2015) in RStudio (RStudio Team, 2021). Compared with repeated measures ANOVAs (RM-ANOVAs), mixed-effects models can deal with unbalanced designs (Jaeger, 2008), such as the current design, with different number of trials in the various conditions.^1^

We ran LMMs with RT as the dependent variable and GLMMs with ACC as the dependent variable. More specifically, the (G)LMMs included the fixed effects of interval (500 ms, 1,500 ms), target location (high-short, high-long, low) and their interaction. In addition, as participants’ awareness of the regularities, intertrial location priming, and the physical location of the target might explain some variance of participants’ performance in the visual search task (Huang et al., 2021), we also included participants’ awareness of spatial regularities (both aware, one aware, and unaware), awareness of the high-probability location after the short interval (aware, unaware), awareness of the high-probability location after the long interval (aware, unaware), intertrial target location priming (yes, no), and physical location of the target on the screen (0–5) as additional fixed effects in our models. All the fixed effects were dummy coded. The random effect structure of the models was determined by running the maximal random effect structure justified by the design which allowed model convergence (Barr et al., 2013). In Experiment 1, for the LMMs, the random effect structure included a by-participant random intercept and a by-participant random slope for target location and interval. For the GLMMs, the random effect structure included a by-participant random intercept and a by-participant random slope for target location. The *p* values were obtained by the likelihood ratio test for all model comparisons in which the model with the fixed effect of interest was compared with the model without it (α = .05). Pairwise tests were investigated using the *emmeans* package with the Tukey correction for multiple comparisons.

### Results

#### RTs

**Fig. 2:**
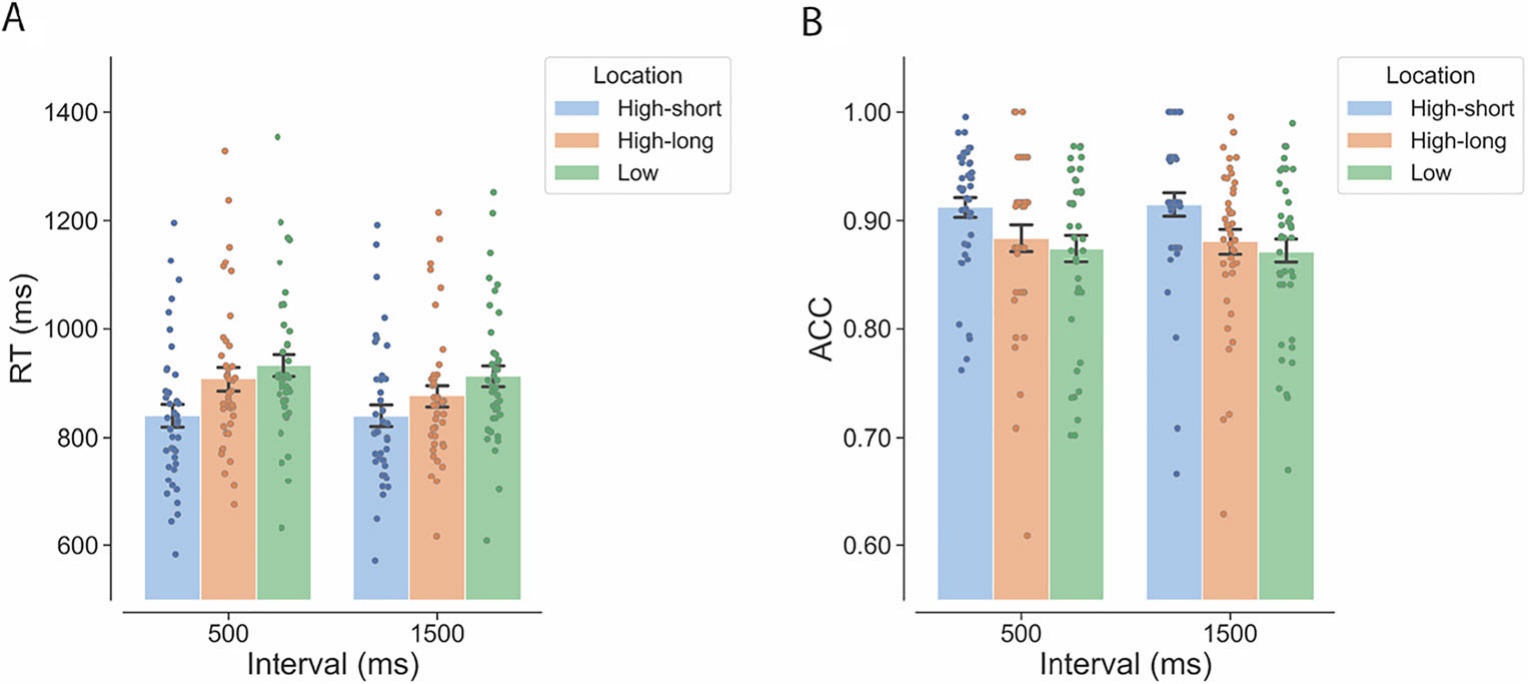
Individual (dots) and overall (bars) mean RT (**A**) and mean ACC (**B**) as a function of the interval and target location in Experiment 1 (uniform distribution of the two intervals). *Note.* RT = reaction time; ACC = accuracy. “High-short” refers to the high-probability target location associated with the short interval. “High-long” refers to the high-probability target location associated with the long interval. “Low” refers to the four low-probability locations. Error bars represent ± 1 between-subjects standard error of the condition means. (Color figure online)

Figure 2A shows the mean RT as a function of interval (500 ms, 1,500 ms) and target location (high-short, high-long, low). As shown in Table 2, the LMM analyses of RTs revealed a significant fixed effect of interval, showing a reduction of RT from the short to the long interval condition. This reflects the effect of general temporal preparation under the uniform interval distribution. There was also a significant fixed effect of target location. Compared with the mean RT for the low-probability target locations, the mean RTs were significantly shorter for the high-short location, β = −77.900, *SE* = 8.360, *t* = −9.319, *p* < .001, and for the high-long location, β = −26.200, *SE* = 9.050, *t* = −2.894, *p* = .017. The mean RT for the high-long location was significantly longer than that for the high-short location, β = 51.700, *SE* = 13.440, *t* = 3.848, *p* = .001.

**Table 2:** Tests of the fixed effects in Experiment 1

	RT (LMMs)			ACC (GLMMs)		
	χ^2^	*df*	*p*	χ^2^	*df*	*p*
Fixed effect						
Interval	7.176	1	.007**	0.090	1	.765
Target location	52.539	2	<.001***	20.012	2	<.001***
Interval × Target location	8.045	2	.018*	0.149	2	.928
Spatial awareness	2.232	2	.328	1.434	2	.488
Spatiotemporal short awareness	0.021	1	.886	0.043	1	.837
Spatiotemporal long awareness	4.282	1	.039*	0.000	1	.999
Target location priming	109.679	1	<.001***	4.066	1	.044*
Physical target position^1^	15.730	5	.008**	20.661	5	<.001***

*Note.* “RT (LMMs)” means the linear mixed models for RT, and “ACC (GLMMs)” means the generalized liner mixed models for accuracy. “Interval × Target location” refers to the interaction between interval and target location. “Spatiotemporal short awareness” refers to the awareness for the high-probability location associated with the short interval, while “Spatiotemporal long awareness” refers to the awareness for the high-probability location associated with the long interval. All the reported chi-squared values for the fixed effects were obtained by the likelihood ratio test for model comparisons in which the model with the fixed effect of interest was compared with the model without it. The models for testing the fixed effect of “Interval” and “Target location” did not include the interaction effect between interval and target location. *p < .05, **p < .01, ***p < .001.

^1^It is noteworthy that we also found a significant effect of the physical location of the target in all experiments. This is consistent with Huang et al. (2021), in which they found the advantages of the specific physical locations in online testing with the current paradigm.

More importantly, the interaction between interval and target location was also significant. Across trials with the 500-ms interval, mean RT was significantly shorter when the target appeared at the high-short location than when it appeared at any one of the low-probability target locations, β = −81.000, *SE* = 8.690, *t* = −9.313, *p* < .001, implying that the temporally congruent location was prioritized compared with the temporally neutral locations at the short interval. In contrast, at the 500-ms interval, there was no significant difference between the temporally incongruent high-long location and the low-probability locations, β = −15.400, *SE* = 11.840, *t* = −1.299, *p* = .399. Also, the mean RT was significantly longer when the target appeared at the temporally incongruent high-long location than when it appeared at the temporally congruent high-short location, β = 65.600, *SE* = 14.980, *t* = 4.378, *p* < .001. In short, there was a response benefit for the temporally congruent high-short location compared with all other locations in the short interval condition.

Across trials with the 1,500-ms interval, participants responded faster when the target appeared at the high-long location relative to the neutral low-probability locations, β = −26.300, *SE* = 9.360, *t* = −2.811, *p* = .020, suggesting a behavioral advantage for the temporally congruent location than the neutral locations at the long interval. Interestingly, the mean RT for the target appearing at the high-short location was also significantly shorter than that for the target appearing at any one of the neutral low-probability locations, β = −60.300, *SE* = 11.280, *t* = −5.347, *p* < .001. The mean RT for the high-long location was not significantly different from that for the high-short location at the 1,500-ms interval, β = 34.000, *SE* = 14.930, *t* = 2.277, *p* = .067. That is, after the long interval, there was a response benefit for both high-probability locations compared with the neutral locations.

#### Accuracy

Figure 2B shows the mean ACC as a function of interval (500 ms, 1,500 ms) and target location (high-short, high-long, low). The GLMM analyses of ACCs only revealed a significant main effect of target location (Table 2). Compared with the mean ACC for the low-probability target locations, the performance for the high-short location was significantly more accurate, β = 0.394, *SE* = 0.080, *z* = 4.947, *p* < .001. There was no significant difference between the high-long location and the low-probability locations, β = 0.108, *SE* = 0.063, *z* = 1.712, *p* = .201. Participants responded less accurately when the target appeared at the high-long location than when it appeared at the high-short location, β = −0.286, *SE* = 0.088, *z* = −3.251, *p* = .003.

#### Awareness assessment

In Experiment 1, a certain number of participants showed awareness for the spatial distribution of targets. Among the 41 participants, 25 participants reported both high-probability target locations correctly. Fourteen participants reported only one high-probability location and two participants reported none of the high-probability locations. There were few participants who showed awareness regarding the spatiotemporal regularities, as only 11 participants reported the associations between the intervals and high-probability locations correctly. Sixteen participants reported none of the spatiotemporal associations. Fourteen participants reported the association between either the short interval (11 participants) or long interval (three participants) with the high-probability target location.

We investigated whether participants’ awareness about the regularities had a significant effect on their performance in the (G)LMM analyses. As shown in Table 2, LMM revealed only a significant effect on RT of the awareness of the high-probability location after the long interval. Participants who were aware of the high-probability target location after the long interval responded faster than those who were unaware of it, β = −86.100, *SE* = 38.700, *t* = −2.225, *p* = .032. In the GLMMs, there was no significant effect on ACC regarding the awareness of regularities.

Furthermore, to determine whether participants’ levels of awareness affected the learning of spatiotemporal regularities, we rated participants’ overall awareness by combing their awareness for the spatial and spatiotemporal regularities. We categorized those participants who correctly reported either the spatial or spatiotemporal regularities as “aware”, while others as “unaware”. Apart from the control factors of target location priming and physical target position reported above, the (G)LMMs included the fixed effects of interval (500 ms, 1,500 ms), target location (high-short, high-long, low), awareness (aware, unaware), and the three-way interaction. For the LMMs, the random effect structure included a by-participant random intercept and a by-participant random slope for target location and interval. For the GLMMs, the random effect structure included a by-participant random intercept and a by-participant random slope for target location and awareness. The results showed that there was no significant interaction between interval, target location and awareness on RT, χ^2^(2) = 1.032, *p* = .597, or on ACC, χ^2^(2) = 4.994, *p* = .082. Therefore, there is no relationship between the level of awareness of the regularities and the effect of those regularities on behavior.

#### Intertrial location priming

As the current findings might also be attributable to short-lasting intertrial location priming (Maljkovic & Nakayama, 1994), we also included “target-location priming” (intertrial target repetition versus alternation) as a fixed effect in the (G)LMMs. The influence of target location priming was significant (Table 2). Participants responded slower, β = 35.800, *SE* = 3.420, *t* = 10.486, *p* < .001, and less accurately, β = −0.097, *SE* = 0.048, *t* = −2.016, *p* = .044, when the target location was not repeated than when it was repeated from the previous trial. However, after considering the effect of target location priming, the behavioral expression of spatiotemporal regularities remained. Thus, the observed effects cannot be explained by intertrial location priming.

In sum, the results of RT revealed an asymmetric time-specific behavioral benefit between two high-probability locations at two intervals. Specifically, there was a response benefit for the temporally congruent high-short location as compared with all other locations at the short interval, while there was a response benefit for both high-probability locations compared with the neutral locations at the long interval. The effects cannot be explained by the participants’ awareness of the regularities or intertrial location priming.

#### The pure effect of spatiotemporal regularities

According to the hypothesis of spatiotemporal modulation of attention, we expected a symmetric temporal benefit at both intervals. That is, we would expect an increase of RT from the 500-ms interval condition to the 1,500-ms interval condition when the target appeared at the high-short location. As we used a uniform distribution of intervals in this experiment, this expectation fails to take into account the counteracting effect of general temporal preparation. In fact, the decrease of RT from the 500-ms interval to the 1,500-ms interval is clearly visible for neutral low-probability locations, β = 20.117, *SE* = 7.360, *t* = 2.734, *p* = .008 (Fig. 2A). When the target appeared at the high-long location, there was also a reduction of RT from the nonassociated 500-ms interval condition to the associated 1,500-ms interval condition, β = 31.044, *SE* = 9.490, *t* = 3.271, *p* = .001. By contrast, when the target appeared at the high-short location, there was no significant difference between the associated 500-ms interval condition and the nonassociated 1,500-ms interval condition, β = −0.547, *SE* = 9.380, *t* = −0.058, *p* = .954. Therefore, the expected increase in RT as a function of interval for the high-short location because of the spatiotemporal regularities might be cancelled out by a concurrent influence of temporal preparation working in the opposite direction.

**Fig. 3:**
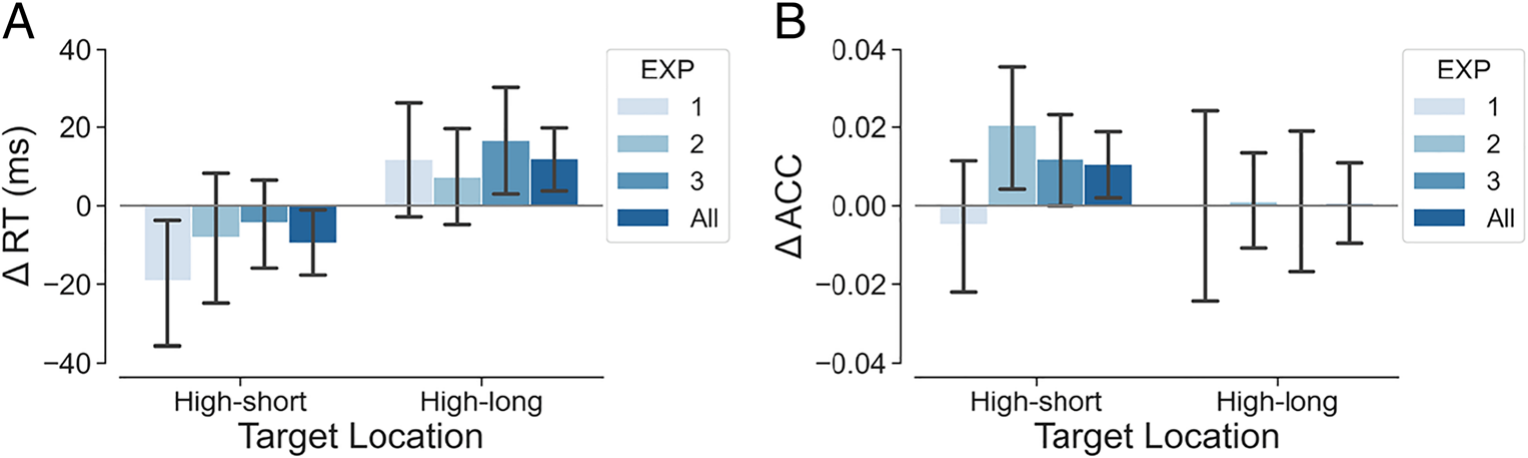
Effect of interval (500 ms minus 1,500 ms) at the two high-probability locations relative to the corresponding baseline effect at the low-probability locations in Experiments 1, 2, 3 and all experiments. *Note.* Δ RT refers to the mean effect of interval (500 ms minus 1,500 ms) at the high-probability locations after subtracting out the corresponding effect of interval obtained at the low-probability locations. Δ ACC refers to the corresponding baseline-corrected effect for accuracy. Error bars represent 95% confidence interval (CI) of the condition means

To assess the pure effect of the spatiotemporal regularities independent of the general temporal preparation, we expressed the effect of interval for the high-short and high-long locations relative to the baseline of this effect for the low-probability locations. In particular, we subtracted out the mean effect of interval (500 ms minus 1,500 ms) obtained at the low-probability locations from the corresponding effect obtained at the high-short location and the high-long location for each participant. Then the effect of interval at these two locations was compared against zero in one-tailed one-sample *t* tests (Fig. 3).

As shown in Fig. 3A, relative to the baseline, the effect of interval on RT was negative for the high-short condition in Experiment 1, *t*(40) = −1.915, *p* = .031, *d* = −0.299. That is, after baseline correction, participants responded faster when the target appeared at the high-short location after the associated short interval than when it appeared at this location after the nonassociated long interval. In comparison, the baseline-corrected effect of interval for the high-long condition was not significant in Experiment 1, but it tended to be positive numerically, *t*(40) = 1.310, *p* = .099, *d* = 0.205. These results indicate that spatiotemporal dynamics influenced performance in addition to general temporal preparation.

### Discussion

Experiment 1 showed that participants learned the spatial distribution of targets at two high-probability locations. Participants responded faster for both the high-short and the high-long location relative to low-probability locations. In addition, there was also evidence for a modest temporal modulation of this effect. Specifically, after the short interval, there was a larger response benefit when the target appeared at the temporally congruent high-short location than when it appeared at any one of the low-probability locations. After the long interval, there was a similar advantage for the temporally congruent high-long location. This finding is consistent with the idea that attention dynamically shifts toward the high-probability location where the target is most likely to appear.

However, this interpretation is complicated by the further finding that, after the long interval, the high-short location continues to have an advantage relative to low-probability locations. This finding seems to suggest that the high-short location continues to be prioritized at the long interval, whereas the high-long location was not yet prioritized at the short interval. Nevertheless, after taking the obscuring influence of general temporal preparation into account, a more symmetrical picture arises. As shown in Fig. 3, in Experiment 1, relative to the baseline of the low-priority location, a long temporal interval leads to a decreased focus of attention at the high-short location and a somewhat increased focus of attention at the high-long location. In Experiments 2 and 3, we further explored the stability of this data pattern under different distributions of intervals. In the general discussion section, we will more deeply consider the implications for the dynamics of attention across space.

In sum, by using the singleton task, we aimed to examine the dynamics of spatial attention separated from motor response learning, which may have contributed to the dynamics in earlier studies (e.g., Boettcher et al., 2022; Wagener & Hoffmann, 2010). The results of Experiment 1 showed that participants are able to implicitly extract the spatiotemporal regularities in the stimulus displays and let those guide attentional search accordingly. This dynamic effect was small, however, and may have been distorted by general temporal preparation, which motivated Experiments 2 and Experiments 3.

## Experiment 2

As revealed in Experiment 1, the effect of spatiotemporal modulation of attention by spatiotemporal regularities might be obscured by a concurrent influence of temporal preparation under a uniform interval distribution. To investigate the pure effect of spatiotemporal regularities of target on visual selection, we adopted the same spatiotemporal regularities in Experiment 2 while employing an exponential distribution of two interval (2:1) to control the effect of general temporal preparation. That is, we made the trials with the 500-ms interval twice as frequent as the trials with the 1,500-ms interval. We expected that the exponential distribution of intervals would result in an approximately flat RT–interval function for neutral locations (cf. Los et al., 2017; Los et al., 2021; Näätänen, 1971; Vangkilde et al., 2013). Furthermore, we expected that, relative to this baseline, the effect of spatiotemporal orienting of visual attention would show a symmetric benefit pattern.

### Method

Based on the effect size of the critical interaction in Experiment 1, a sample size of 54 participants was suggested by the power analysis. To achieve the aimed sample size in the final data sample considering the exclusion of participants, we recruited fifty-seven participants for credits on SONA in this experiment. None of the participants had participated in Experiment 1. One participant was excluded because of low accuracy (less than 70%). The final sample included 56 participants (age 21.04 ± 5.65 years, 43 females, one other gender). The apparatus was identical to that of Experiment 1. The procedure and design were the same as in Experiment 1, with the exception that the distribution of two intervals was exponential (500 ms : 1,500 ms = 2:1). The experiment consisted of a practice block of 25 trials and 10 experimental blocks of 84 trials each (shown in Table 1).

Trials with RT below 200 ms or above 2000 ms (2.07% of all trials) were excluded. Also, for RT analysis, incorrect trials (9.03% of all trials) and trials with RTs outside ± 2.5 standard deviation of the condition mean for each participant (2.36% of all trials) were excluded. The filtered data were entered in the (G)LMMs. In Experiment 2, the model specification of (G)LMMs were the same as in Experiment 1.

### Results

#### RTs

**Fig. 4:**
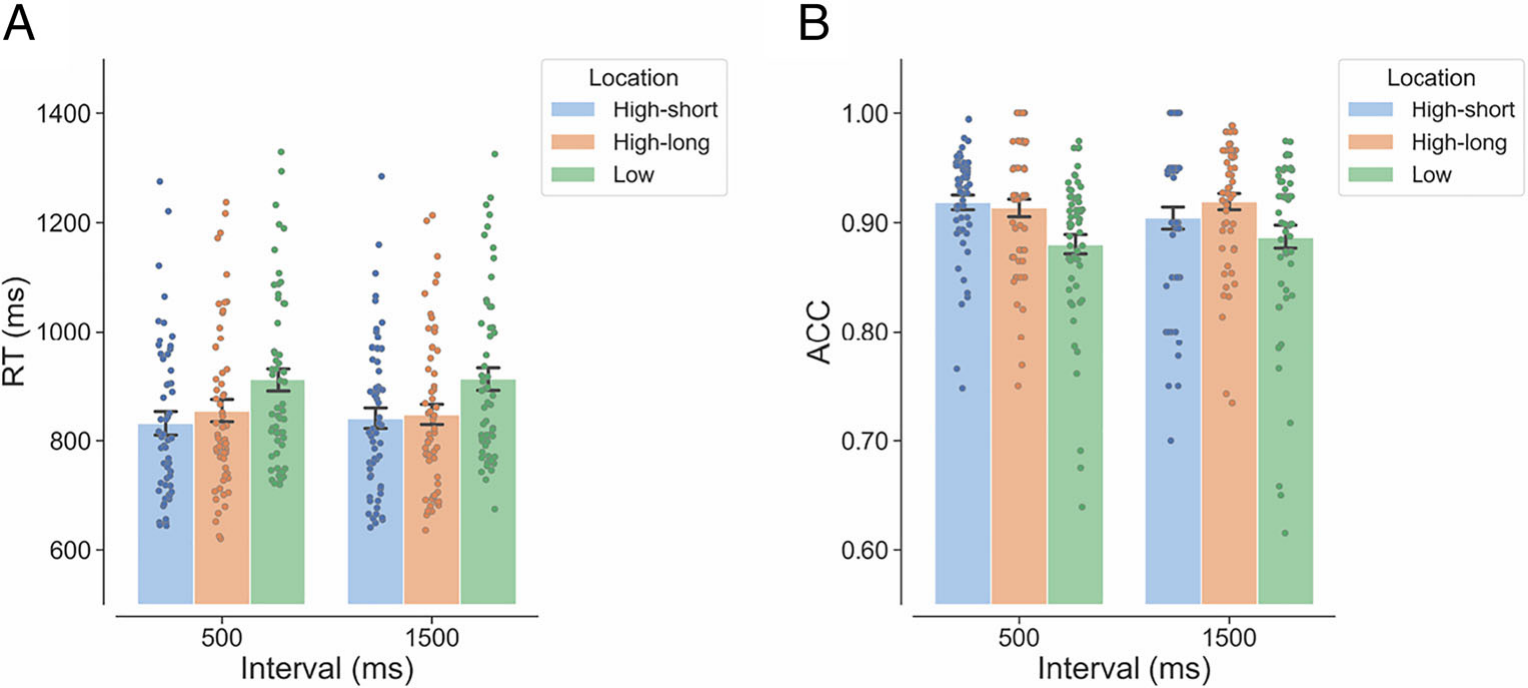
Individual (dots) and overall (bars) mean RT (**A**) and mean ACC (**B**) as a function of the interval and target location in Experiment 2 (exponential distribution of two intervals). *Note.* RT = reaction time; ACC = accuracy. “High-short” refers to the high-probability target location associated with the 500-ms interval. “High-long” refers to the high-probability target location associated with the 1,500-ms interval. “Low” refers to the four low-probability locations. Error bars represent ± 1 between-subjects standard error of the condition means. (Color figure online)

Figure 4A shows the mean RT as a function of interval (500 ms, 1,500 ms) and target location (high-short, high-long, low) in Experiment 2. As shown in Table 3, the LMM analyses of RTs showed no significant main effect of interval, revealing an approximately flat RT–interval function in all the locations. This indicates that the manipulation of interval distribution worked as expected. There was a significant fixed effect of target location. Compared with the mean RT for the low-probability target locations, the mean RTs were significantly shorter for the high-short location, β = −68.400, *SE* = 7.820, *t* = −8.746, *p* < .001, and for the high-long location, β = −57.100, *SE* = 6.970, *t* = −8.194, *p* < .001. There was no significant difference between the high-long location and the high-short location, β = 11.300, *SE* = 8.760, *t* = 1.293, *p* = .406. Contrary to the results of Experiment 1, the interaction between interval and target location was not significant.

**Table 3:** Tests of the fixed effects in Experiment 2

	RT (LMMs)			ACC (GLMMs)		
	χ^2^	*df*	*p*	χ^2^	*df*	*p*
Fixed effect						
Interval	0.000	1	.997	0.439	1	.507
Target location	60.879	2	<.001***	30.091	2	<.001***
Interval × Target location	3.367	2	.186	4.580	2	.101
Spatial awareness	0.143	2	.931	3.798	2	.150
Spatiotemporal short awareness	7.526	1	.006**	1.033	1	.309
Spatiotemporal long awareness	2.130	1	.144	1.291	1	.256
Target location priming	133.273	1	<.001***	6.118	1	.013*
Physical target position	21.513	5	<.001***	19.786	5	.001**

*Note.* “RT (LMMs)” means the linear mixed models for RT, and “ACC (GLMMs)” means the generalized liner mixed models for accuracy. “Spatiotemporal short awareness” refers to the awareness for the high-probability location associated with the short interval, while “Spatiotemporal long awareness” refers to the awareness for the high-probability location associated with the long interval. All the reported chi-squared values for the fixed effects were obtained by the likelihood ratio test for all model comparisons in which the model with the fixed effect of interest was compared with the model without it. The models for estimating the fixed effect of “Interval” and “Target location” did not include the interaction effect between interval and target location. *p < .05, **p < .01, ***p < .001

#### Accuracy

Figure 4B shows the mean ACC as a function of interval (500 ms, 1,500 ms) and target location (high-short, high-long, low) in Experiment 2. The GLMM analyses of the ACC data revealed no significant effect of interval but a significant effect of target location (Table 3). Compared with the mean ACC for the low-probability target locations, the mean ACCs were both significantly higher for the high-short location, β = 0.358, *SE* = 0.068, *z* = 5.237, *p* < .001, and for the high-long location, β = 0.431, *SE* = 0.079, *z* = 5.442, *p* < .001. There was no significant difference between the high-long location and the high-short location, β = 0.073, *SE* = 0.080, *z* = 0.906, *p* = .637. The interaction between interval and target location was not significant.^2^

#### Awareness assessment

Among the 56 participants, 33 participants reported both two high-probability locations correctly. Twenty participants reported only one high-probability location correctly and three participants reported neither of the high-probability locations correctly. Most of the participants were unaware of the spatiotemporal regularities, as 31 participants reported neither of the associations between intervals and high-probability locations. Seven participants reported the associations between both intervals and high-probability locations correctly. Eighteen participants reported the association between either the short interval (12 participants) or the long interval (six participants) with the high-probability target location correctly.

To investigate whether there was a significant effect of participants’ awareness, we included awareness as fixed factors in the (G)LMM analyses (Table 3). There was only a significant effect of awareness of the high-probability location after the short interval on RT. However, being aware of the high-probability location after the short interval slowed down response, β = 109.000, *SE* = 35.100, *t* = 3.120, *p* = .003. In the GLMMs, there was no significant effect regarding awareness of the regularities. Thus, the results did not provide any evidence that the effects were dependent on participants being aware of the corresponding contingencies.

Also, we examined the relationship between the level of awareness of the regularities and the size of learning in the (G)LMMs by including the fixed effects of interval (500 ms, 1,500 ms), target location (high-short, high-long, low), awareness (aware, unaware), and the three-way interaction apart from the intertrial target location priming and physical target location. For the LMMs, the random effect structure included a by-participant random intercept and a by-participant random slope for target location, interval, and awareness. For the GLMMs, the random effect structure included a by-participant random intercept and a by-participant random slope for target location and awareness. The results showed no significant interaction among interval, target location and awareness on RT, χ^2^(2) = 0.151, *p* = .927, or on ACC, χ^2^(2) = 1.418, *p* = .492.

#### Intertrial location priming

As shown in Table 3, the influence of target location priming was significant. Participants responded slower, β = 29.100, *SE* = 2.520, *t* = 11.554, *p* < .001, and less accurately, β = −0.101, *SE* = 0.041, *t* = −2.476, *p* = .013, when the target location was non-repeated than when it was repeated from the previous trial. However, after controlling for target location priming in the (G)LMMs, the learning effect reported earlier remained. Thus, these effects were not attributable to short lasting intertrial location priming.

In sum, the results showed that both high-probability locations were prioritized over the low-probability locations regardless of the interval. The effects cannot be explained by awareness of the regularities or intertrial target location priming.

#### The pure effect of spatiotemporal regularities

Although there was no significant effect of interval in any of the location conditions under the exponential distribution, we again examined the pure effect of spatiotemporal regularities by subtracting out the mean effect of interval obtained at the low-probability locations from the corresponding effect obtained at the high-short and the high-long locations for each participant. Then the baseline-corrected effect of interval at two high-probability locations was again compared against zero in one-tailed one-sample *t* tests. As shown in the Fig. 3B, there was a positive effect of interval on ACC at the high-short location after controlling for the baseline effect. Participants responded more accurately when the target appeared at the high-short location after the associated short interval than after the nonassociated long interval, *t*(55) = 2.138, *p* = .018, *d* = 0.286. This finding indicates that spatiotemporal regularities influenced visual attention. However, we observed no corresponding benefit at the high-long location, *t*(55) = 0.135, *p* = .553, *d* = 0.018. The corresponding analysis on RT yielded a similar trend as Experiment 1 (Fig. 3A), but the effect was not significant for either interval (*p*s > .1).

### Discussion

In Experiment 2, we obtained an approximately flat RT–interval function, indicating that the application of the exponential distribution of intervals worked as intended. With the same spatiotemporal distribution of targets as in Experiment 1, we found again a general prioritization of high-probability locations on RT but without the temporal dynamics. After both intervals, participants responded faster when the target was presented at either one of the high-probability locations relative to low-probability locations. Contrary to the findings of Experiment 1, this spatial benefit was not modified by temporal interval. However, numerically, the expression of spatiotemporal regularities was still present, both on RT and accuracy, which comes to the fore most clearly in the baseline corrected data (Fig. 3). Moreover, in the baseline corrected analyses, it turned out that the response accuracy for the high-short location (though not for the high-long location), was significantly higher after the associated short interval than after the nonassociated long interval (Fig. 3B). So, overall, Experiment 2 revealed some spatiotemporal effect, although not as convincingly as in Experiment 1, perhaps because it was partially transferred from RT to accuracy.

In sum, after controlling for the potential influence of general temporal preparation, Experiment 2 also revealed some evidence that participants implicitly learn the spatiotemporal regularities, and that attentional search was at least partially guided by it. However, this effect was very modest, and seemed to have partially shifted from RT (Experiment 1) to accuracy (Experiment 2) for unclear reasons. It is also unclear why this effect was asymmetric on ACC, being significant for the high-short location but absent for the high-long location. Regarding the latter, one reason could be that, in absolute terms, the high-short location was presented much more frequently than the high-long location in Experiment 2 (cf. Table 1).

## Experiment 3

The evidence so far suggests that spatiotemporal regularities can be implicitly learned and guide attentional orienting dynamically in space. However, the behavioral expression of the spatiotemporal regularities was not very strong and revealed some differences between Experiments 1 and 2. In particular, in Experiment 2, the high-long location occurred less frequently than the high-short location in an absolute sense, which may explain that it did not reveal any spatiotemporal dynamics. Therefore, we performed yet another test of our hypothesis in Experiment 3 by adopting the anti-exponential distribution of intervals, in which the number of trials with the long interval was twice the number of trials with a short interval. In previous choice RT tasks, this distribution yields a relatively steep RT–interval function (Los et al., 2017; Los et al., 2021). Accordingly, under the anti-exponential interval distribution of Experiment 3, the most frequent target location was the high-long location. If the absolute frequency of target locations is an important determinant of spatiotemporal dynamics, we expect to see these dynamics for the high-long location but not for the high-short location.

### Method

We aimed for a calculated sample size of 60 participants based on the effect size of Experiment 2. To achieve the aimed sample size in the final data sample considering the exclusion of participants, 64 participants were recruited to participate in this experiment for credits. None of the participants had participated in Experiments 1 or 2. Three participants were excluded because of low accuracy (less than 70%), and two participants were excluded because they took at least 4 hours to complete the experiment. Data are presented for 59 participants (age 21.76 ± 3.72 years, 33 females, 5 other genders). The apparatus was identical to that of Experiment 2. The design of Experiment 3 was the same as that of Experiment 2, except that the distribution of two intervals was anti-exponential (500 ms: 1,500 ms = 1: 2; see Table 1).

Trials with RT below 200 ms or above 2000 ms (2.13% of all trials) were excluded. For the analysis of RT, incorrect trials (9.37% of all trials) and trials with RTs outside ± 2.5 standard deviation of the condition mean for each participant (2.39 % of all trials) were also excluded. The filtered data were entered in (G)LMMs. In Experiment 3, the model specification of both LMMs and GLMMs were the same as in Experiments 1 and 2.

### Results

#### RTs

**Fig. 5:**
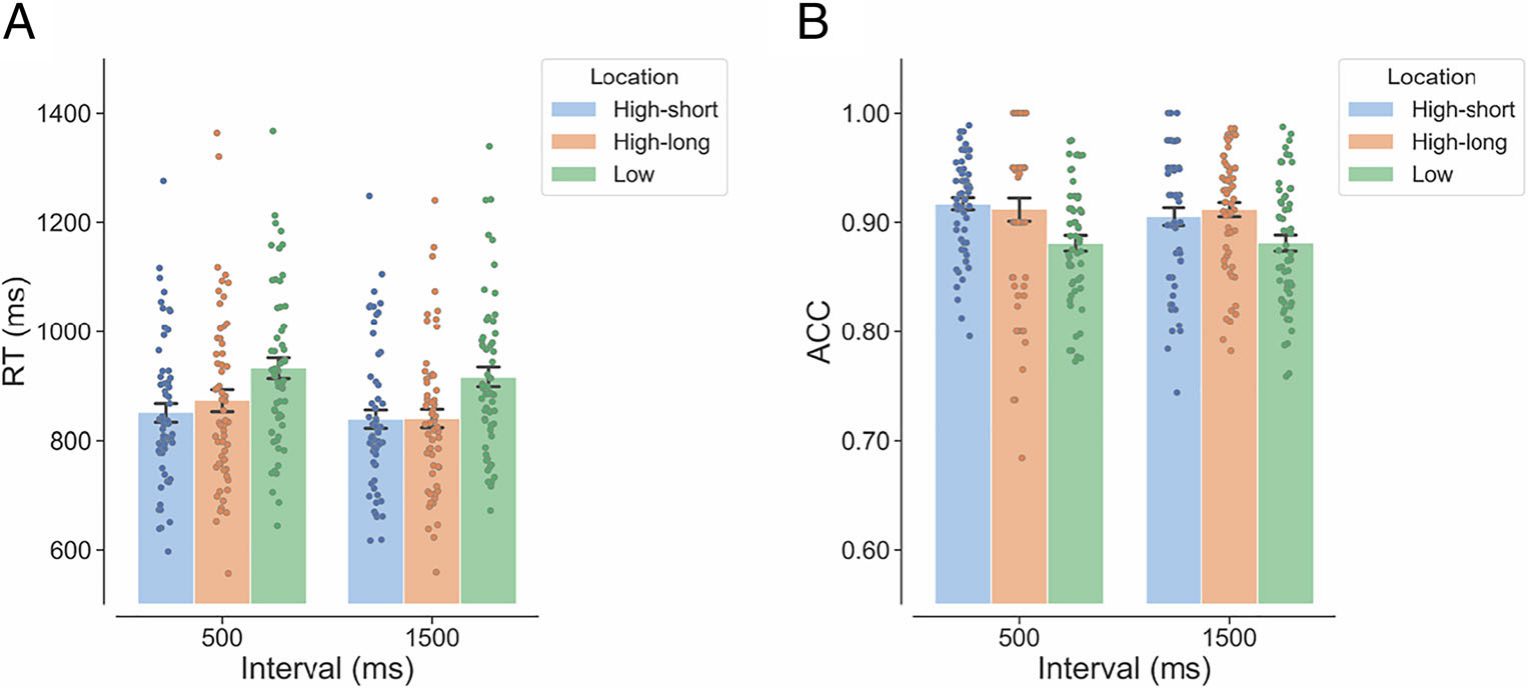
Individual (dots) and overall (bars) mean RT (**A**) and mean ACC (**B**) as a function of the interval and target location in Experiment 3 (anti-exponential distribution of two intervals). *Note.* RT = reaction time; ACC = accuracy. “High-short” refers to the high-probability target location associated with the 500-ms interval. “High-long” refers to the high-probability target location associated with the 1,500-ms interval. “Low” refers to the four low-probability locations. Error bars represent ± 1 between-subjects standard error of the condition means. (Color figure online)

Figure 5A shows the mean RT as a function of interval (500 ms, 1,500 ms) and target location (high-short, high-long, low) under an anti-exponential distribution of the two intervals. As shown in Table 4, the LMM analyses of the RTs revealed a significant fixed effect of interval, revealing a strong reduction of RT from the short interval condition to the long interval condition. This is typical for the anti-exponential interval distribution, which suggests that the manipulation of interval distribution was successful. There was also a significant effect of target location. Compared with the mean RT for the low-probability target locations, the mean RTs for the high-short location, β = −73.200, *SE* = 6.340, *t* = −11.542, *p* < .001, and for the high-long location, β = −60.900, *SE* = 9.130, *t* = −6.673, *p* < .001, were both significantly shorter. There was no significant difference between the high-long location and the high-short location, β = 12.300, *SE* = 8.900, *t* = 1.380, *p* = .358. Most importantly, the interaction between interval and target location was significant.

**Table 4:** Tests of the fixed effects in Experiment 3

	RT (LMMs)			ACC (GLMMs)		
	χ^2^	*df*	*p*	χ^2^	*df*	*p*
Fixed effect						
Interval	14.734	1	<.001***	0.825	1	.364
Target location	71.180	2	<.001***	31.192	2	<.001***
Interval × Target location	6.684	2	.035*	2.419	2	.298
Spatial awareness	3.437	2	.179	1.105	2	.576
Spatiotemporal short awareness	0.018	1	.894	0.026	1	.872
Spatiotemporal long awareness	0.010	1	.922	0.857	1	.355
Target location priming	226.136	1	<.001***	17.250	1	<.001***
Physical target position	18.441	5	.002**	41.344	5	<.001***

*Note.* “RT (LMMs)” means the linear mixed models for RT, and “ACC (GLMMs)” means the generalized liner mixed models for accuracy. “Spatiotemporal short awareness” refers to the awareness for the high-probability location associated with the short interval, while “Spatiotemporal long awareness” refers to the awareness for the high-probability location associated with the long interval. All the reported chi-squared values for the fixed effects were obtained by the likelihood ratio test for all model comparisons in which the model with the fixed effect of interest was compared with the model without it. The models for estimating the fixed effect of “Interval” and “Target location” did not include the interaction effect between interval and target location. *p < .05, **p < .01, ***p < .001

When the search display was presented after the 500-ms interval, participants responded significantly faster when the target appeared at the temporally congruent high-short location than when it appeared at any of the neutral low-probability target locations, β = −73.200, *SE* = 6.840, *t* = −10.701, *p* < .001. The mean RT for the high-long location was also significantly faster than that for the low-probability locations, β = −45.100, *SE* = 11.510, *t* = −3.921, *p* < .001. However, the mean RT for the high-long location was significantly longer than that for the high-short location, β = 28.000, *SE* = 10.820, *t* = 2.591, *p* = .029. Thus, both high-probability locations were prioritized over the low-probability locations, but the behavioral benefit for the temporally congruent high-probability location was bigger than the temporally incongruent one at the short interval.

When the search display was presented after the 1,500-ms interval, participants responded faster if targets appeared at either the high-short location, β = −68.700, *SE* = 7.660, *t* = −8.963, *p* < .001, or appeared at the high-long location, β = −62.400, *SE* = 9.210, *t* = -6.770, *p* < .001, compared with the low-probability locations. The mean RTs for the high-long location and the high-short location were not significantly different, β = 6.300, *SE* = 9.630, *t* = 0.654, *p* = .790. Therefore, both high-probability locations were prioritized over the low-probability locations at the long interval.

#### Accuracy

Figure 5B shows the mean ACC as a function of interval (500 ms, 1,500 ms) and target location (high-short, high-long, low) in Experiment 3. The GLMM analyses of the ACC data only revealed a significant effect of target location (Table 4). Participants were more accurate when the target appeared at either the high-short location, β = 0.361, *SE* = 0.063, *z* = 5.754, *p* < .001, or the high-long location, β = 0.358, *SE* = 0.069, *z* = 5.167, *p* < .001, than when it appeared at any one of the low-probability target locations. There was no significant difference between the high-long location and the high-short location, β = −0.003, *SE* = 0.065, *z* = −0.045, *p* = .999.

#### Awareness assessment

Among 59 participants, 36 participants were able to report both high-probability locations. There were 22 participants who reported only one high-probability location correctly and one participant reported neither of the high-probability locations. Few participants were aware of the spatiotemporal regularities, as only five participants reported both the associations between intervals and high-probability locations correctly and 38 participants reported neither of the associations correctly. Sixteen participants reported the association between either the short interval (nine participants) or the long interval (seven participants) with the high-probability location correctly.

To investigate the effect of participants’ awareness, we again included awareness as fixed factor in the (G)LMM analyses. There was no significant effect related to awareness (Table 4). Even so, we again examined the effects of interval (500 ms, 1,500 ms), target location (high-short, high-long, low), awareness (aware, unaware), and the three-way interaction in the (G)LMMs apart from intertrial target location priming and physical target location. For the (G)LMMs, the random effect structure included a by-participant random intercept and a by-participant random slope for target location and awareness. The results showed that there was no significant interaction between interval, target location and awareness on RT, χ^2^(2) = 0.858, *p* = .651, or on ACC, χ^2^(2) = 0.024, *p* = .988. Therefore, there was no evidence that the effects were modulated by participants’ awareness of the regularities.

#### Intertrial location priming

Consistent with previous experiments, participants responded slower, β = 36.900, *SE* = 2.450, *t* = 15.059, *p* < .001, and less accurately, β = −0.161, *SE* = 0.039, *t* = −4.142, *p* <.001, when the target location was non-repeated than when it was repeated from the previous trial. After controlling for the target location priming in the (G)LMMs, the behavioral expression of spatiotemporal regularities remained. Thus, the observed effects were not attributable to intertrial location priming.

In sum, the results of RT revealed the prioritization of both high-probability locations compared with low-probability locations, while there was a bigger response benefit for the temporally congruent high-short location as compared with the incongruent high-long location at the short interval. In contrast, both high-probability locations were prioritized comparable relative to the low-probability locations at the long interval. The effects cannot be explained by the participants’ awareness of the regularities or intertrial location priming.

#### The pure effect of spatiotemporal regularities

Under an anti-exponential interval distribution, there was a significant difference between the two intervals for the low-probability target locations, β = 16.800, *SE* = 5.410, *t* = 3.101, *p* = .002, and the high-long location, β = 34.000, *SE* = 7.670, *t* = 4.430, *p* < .001, showing a shorter RT for the long interval condition compared with the short interval condition at these locations. There was also a trend for the reduction of RT from the 500-ms condition to the 1,500-ms condition at the high-short location, β = 12.300, *SE* = 6.290, *t* = 1.950, *p* = .053. Again, to explore the pure effect of the spatiotemporal regularities apart from the general temporal expectation under the anti-exponential interval distribution, we excluded the baseline effect of interval obtained at the low-probability location from the corresponding effect at the two high-probability locations. Then we examined the baseline-corrected effect of interval at these two locations by comparing it against zero in one-tailed one-sample *t* tests.

As shown in Fig. 3A, relative to the baseline, the effect of interval for the high-short condition was not significant on RT, *t*(58) = −0.623, *p* = .268, *d* = −0.081. In comparison, the effect of interval was positive for the high-long condition on RT, *t*(58) = 1.937, *p* = .029, *d* = 0.252. Participants responded slower when the target appeared at the high-long location after the nonassociated short interval than when it appeared at this location after the associated long interval. In addition, there was also a positive effect of interval for the high-short location on ACC (Fig. 3B), *t*(58) = 1.681, *p* = .049, *d* = 0.219, indicating more accurate response for the associated short interval condition than the nonassociated long interval condition when the target appeared at the high-short location. In sum, we observed better search performance when the target appeared at the high-probability location after an associated interval than after the nonassociated interval both for the high-short location (on ACC) and for the high-long location (on RT).

### Discussion

After adopting an anti-exponential distribution of two intervals, we observed the expected larger effect of interval in Experiment 3, indicating that the manipulation of the time course of temporal preparation was successful. Again, we found that two high-probability locations were prioritized relative to the low-probability locations at both intervals. Participants responded faster and more accurately when the target appeared at any of the high-probability locations than when it appeared at any of the low-probability locations for both intervals. In addition, in Experiment 3, there was also evidence for spatiotemporal modulation of visual attention. After the short interval, participants tended to respond faster when the target was presented at the temporally congruent high-short location than when it was presented at the temporally incongruent high-long location (Fig. 5A). These findings could not be explained by participants’ awareness of the regularities or by intertrial priming. Besides, after controlling for the effect of temporal preparation obtained at the neutral locations, performance was better, either on ACC or on RT, when the target occurred at the high-probability location after its associated interval than when it occurred there after its nonassociated interval (cf. Fig. 3). Given that targets occurred much less often at the high-short location than at the high-long location in Experiment 3, the absolute frequency of these locations seems not to be an important determinant of the spatiotemporal dynamics of this effect.

## The effect across experiments

Although we observed evidence for spatiotemporal modulation of visual attention in all experiments, there exists some variability for the effect across experiments in different measures (RT/ACC) and conditions (short/long intervals). Therefore, we tested whether the variation in effects across experiments is reliable. To this end, we aggregated and analyzed the data from the three experiments. Apart from all the control factors reported above,^3^ the (G)LMMs included the fixed effects of interval (500 ms, 1,500 ms), target location (high-short, high-long, low), Experiment (EXP1, EXP2, EXP3), and the three-way interaction. For the LMMs, the random effect structure included a by-participant random intercept and a by-participant random slope for target location and interval. For the GLMMs, the random effect structure included a by-participant random intercept and a by-participant random slope for target location. The results revealed no significant interaction among interval, target location and experiment on RT, χ^2^(4) = 2.773, *p* = .597, or on ACC, χ^2^(4) = 3.047, *p* = .550.

Furthermore, we examined the critical interaction between interval and target location across experiments in (G)LMMs, with “Experiment” as an additional fixed effect apart from other control factors. For the LMMs, the random effect structure included a by-participant random intercept and a by-participant random slope for target location and interval. For the GLMMs, the random effect structure included a by-participant random intercept and a by-participant random slope for target location. As shown in Table 5, there was no significant difference across experiments on either RT or on ACC. The GLMMs of ACC revealed no significant interaction between the interval and target location across experiments. In contrast, the LMMs showed a significant interaction between the interval and target location across experiments on RT.

**Table 5:** Tests of the fixed effects across experiments

	RT (LMMs)			ACC (GLMMs)		
	χ^2^	*df*	*p*	χ^2^	*df*	*p*
Fixed effect						
Interval	14.648	1	<.001***	0.122	1	.727
Target location	174.690	2	<.001***	79.837	2	<.001***
Interval × Target location	13.340	2	.001**	4.080	2	.130
Experiment	0.520	2	.771	0.091	2	.956
Spatial awareness	2.391	2	.303	6.017	2	.049*
Spatiotemporal short awareness	1.835	1	.176	0.945	1	.331
Spatiotemporal long awareness	0.020	1	.888	0.018	1	.893
Target location priming	468.450	1	<.001***	24.653	1	<.001***
Physical target position	37.067	5	<.001***	69.159	5	<.001***

*Note.* “RT (LMMs)” means the linear mixed models for RT, and “ACC (GLMMs)” means the generalized liner mixed models for accuracy. “Spatiotemporal short awareness” refers to the awareness for the high-probability location associated with the short interval, while “Spatiotemporal long awareness” refers to the awareness for the high-probability location associated with the long interval. All the reported chi-squared values for the fixed effects were obtained by the likelihood ratio test for all model comparisons in which the model with the fixed effect of interest was compared with the model without it. The models for estimating the fixed effect of “Interval” and “Target location” did not include the interaction effect between interval and target location. *p < .05, **p < .01, ***p < .001

After the short interval, the response was faster when the target appeared at either the high-short location, β = −72.800, *SE* = 4.510, *t* = −16.137, *p* < .001, or the high-long location, β = −40.700, *SE* = 6.160, *t* = −6.609, *p* < .001, compared with when it appeared at any of the low-probability locations. Moreover, participants responded significantly slower when the target appeared at the temporally incongruent high-long location than when it appeared at the temporally congruent high-short location, β = 32.100, *SE* = 6.750, *t* = 4.758, *p* < .001. After the long interval, relative to the neutral low-probability locations, participants responded faster when the target appeared at either the high-long location, β = −51.600, *SE* = 5.200, *t* = −9.934, *p* < .001, or the high-short location, β = −64.300, *SE* = 5.580, *t* = −11.528, *p* < .001. There was no significant difference between the high-long location and the high-short location at the long interval, β = 12.700, *SE* = 6.740, *t* = 1.886, *p* = .145.

In short, there was a response benefit for both the high-short location and the high-long location compared with neutral low-probability locations at the short interval, but the benefit for the temporally congruent high-short location was bigger than that for the incongruent high-long location. At the long interval, there was a comparable response benefit for both high-probability locations compared with the neutral locations. These results again cannot be explained by participants awareness of the regularities or intertrial priming. Overall, we observed the dynamics across three experiments on RT, and we found no evidence for a modifying role of experiment.

### The overall pure effect of spatiotemporal regularities

Also, we examined whether the pure effect of spatiotemporal regularities varies across experiments (Fig. 3). To this end, we subtracted out the mean effect of interval obtained at the low-probability locations from the corresponding effect obtained at the high-short and the high-long locations for each participant from each experiment. Then the baseline-corrected data were entered in LMMs with target location (high-short, high-long), Experiment (EXP1, EXP2, EXP3) and their interaction as fixed effects. The random effect structure included a by-participant random intercept. The analyses revealed no significant interaction between target location and experiment on either ΔRT, χ^2^(2) = 1.078, *p* = .583, or on Δ ACC, χ^2^(2) = 2.209, *p* = .332.

Moreover, we examined the overall pure effect of spatiotemporal regularities across experiments. The baseline-corrected effect of interval at two high-probability locations was again compared against zero in one-tailed one-sample *t* tests. As shown in the Fig. 3A, across experiments, there was a negative effect of interval on RT at the high-short location after controlling for the baseline effect. Participants responded faster when the target appeared at the high-short location after the associated short interval than after the nonassociated long interval, *t*(155) = −1.856, *p* = .033, *d* = −0.149. In contrast, we observed a positive effect of interval on RT at the high-long location. Participants responded slower when the target appeared at the high-long location after the nonassociated short interval than after the associated long interval, *t*(155) = 2.486, *p* = .007, *d* = 0.199. The corresponding analysis on ACC yielded a positive effect of interval at the high-short location (Fig. 3B). Participants responded more accurately when the target appeared at the high-short location after the associated short interval than after the nonassociated long interval, *t*(155) = 2.077, *p* = .020, *d* = 0.166. But the effect of interval on ACC was not significant at the high-long location, *t*(155) = 0.079, *p* = .531, *d* = 0.006.

In sum, after controlling the effect of general temporal preparation, we observed better search performance when the target appeared at a high-probability location after its associated interval than after the nonassociated interval across experiments, revealing the behavioral expression of spatiotemporal modulation of visual attention. Again, we found no evidence for a modifying role of experiment on this effect.

## General discussion

In the current study, we associated two intervals with two high-probability target locations. Consistent with studies on SL of spatial regularities (Druker & Anderson, 2010; Failing et al., 2019), we found that participants learned to prioritize two high-probability target locations relative to low-probability locations. That is, participants responded faster or more accurately when the target appeared at one of the high-probability locations compared with when it appeared at any of the low-probability locations. Going beyond this, we obtained evidence that the prioritization of high-probability target locations is at least partially dynamic. All experiments revealed an interaction between interval and target location on either RT or accuracy. This interaction indicates that participants prioritize high-probability locations depending on when the target is most likely to appear there.

The possibility of extracting the statistical regularities from two remote locations simultaneously through SL has been observed in previous studies (Druker & Anderson, 2010; Failing et al., 2019). At first sight, this finding seems to indicate that spatial attention can be simultaneously divided over several remote areas in space. This would argue against the spotlight metaphor (Posner et al., 1980) in which attention can be focused at only one spatial location at the time. Instead, these findings seem to argue for a view that attention is more malleable in its distribution across space (Cave et al., 2010; Jans et al., 2010; Logan, 1996; McMains & Somers, 2004).

However, in our study, we found some modest evidence for the dynamic allocation of attention towards the temporally congruent high-probability location at a given time point. These dynamics were most obvious in Experiment 1, where only the temporally congruent high-short location was prioritized compared with all other locations at the short interval, while both the temporally congruent high-long location and the temporally incongruent high-short location were prioritized compared with the neutral locations at the long interval (Fig. 2A). Similar, though less evident dynamics were observed in Experiments 2 (numerically) and 3 (on RT). Although there were some differences of the behavioral expression of the spatiotemporal regularities among experiments, in our analysis in which we aggregated the data across experiments, we found no evidence for a modifying role of experiment on either RT or ACC, and the dynamics exists across experiments on RT. Specifically, compared with neutral low-probability locations, there was more prioritization of the temporally congruent high-short location than the temporally incongruent high-long location at the short interval, while there was a comparable prioritization for both high-probability locations compared with the neutral locations at the long interval. After controlling for the baseline effect of temporal preparation, we observed behavioral advantages when the target appeared at the high-probability location after its associated interval as compared with when it appeared at that location after its nonassociated interval. As Fig. 3 shows, these dynamics were present in all three experiments.

When applied to the present findings, the dynamic mechanism implies that, after the onset of the neutral fixation dot, attention is first allocated to the high-probability location associated with the short interval. If the target is presented there, this results in a relative behavioral benefit for the temporally congruent high-short location compared with the temporally incongruent high-long location (Figs. 2 and 5). In trials with the long interval, attention switches towards the high-long location when no search display was presented after 500 ms, leading to a behavioral benefit when the target appeared at the newly attended location. However, this mechanism does not explain that, after the long interval in the present experiments, the high-short location continued to have a behavioral benefit over the low-probability locations (see Xu et al., 2021, for a similar finding in the context of distractor suppression). To accommodate this finding, one could assume that, at least on some trials or in some participants, attention lingers at the high-short location until the end of the long interval, and only moves towards the high-long location after inspecting the high-short location. This mechanism of lingering attention would result in the behavioral pattern of prioritization of two locations. This assumption is supported by evidence that attention can be directed to a new location before it is entirely disengaged from its previous locus (Gabbay et al., 2019). However, several alternative mechanisms may underlie the dynamic pattern observed in the current study, and without additional information, these alternatives cannot be distinguished. Future studies could therefore further use eye tracking (see also Pfeuffer et al., 2020) or EEG to clarify the exact mechanism behind the current findings.

Disregarding the exact mechanism of attention allocation, the current findings are likely an expression of SL of the spatiotemporal regularities of the target. First, the observed effects were not attributable to participants’ explicit awareness of the regularities. Although many participants showed awareness of the spatial distribution of targets, and some of them even of the spatiotemporal regularities, it turned out that the participant’s level of awareness did not account for the findings in any one of the three experiments. There was also no evidence that the participant’s level of awareness of the regularities affects the SL of spatiotemporal regularities. This is consistent with the findings from previous studies about SL of spatial regularities (Geng & Behrmann, 2002; Wang et al., 2019; Wang & Theeuwes, 2018a) or temporal regularities (Li & Theeuwes, 2020; Los et al., 2017; Los et al., 2021; Olson & Chun, 2001; Thomaschke & Dreisbach, 2015; Wagener & Hoffmann, 2010). Second, the findings cannot be explained by intertrial location priming. The behavioral expression remained in place in all three experiments after controlling the effect of intertrial location priming in (G)LMMs. Similarly, previous studies have also shown that the behavioral adaption to statistical regularities exists after excluding repetition trials from data analyses (Li & Theeuwes, 2020; Wang et al., 2019; Wang & Theeuwes, 2018a), or after removing the regularities in a subsequent test phase (Mattiesing et al., 2017; Rieth & Huber, 2013; Thomaschke & Dreisbach, 2015). Therefore, the current study supports the idea that SL plays an important role in attentional control apart from top-down and bottom-up mechanisms (Awh et al., 2012; Theeuwes, 2019).

Finally, it should be noted that in the current study we did not control for eye movements in a strict way. Thus, it is possible that (small) eye movements did play a role in obtaining the current effects. Yet, by using the same paradigm, previous studies have demonstrated that people can learn the spatial regularities while covertly attended target and distractor locations, since eye tracking was used to avoid systematic eye movements (van Moorselaar et al., 2021; Wang & Theeuwes, 2018a). Therefore, it is reasonable to assume that spatiotemporal regularities can be learned when participants covertly attend target locations.

In conclusion, the present study showed that SL of spatiotemporal regularities may come to dynamically guide visual attention across space even after controlling for a (strong) contribution of motor learning. Attention was dynamically oriented toward the probable target locations according to the statistical regularities of target distribution across space and time. Also, the learning of spatiotemporal regularities was independent from the general temporal preparation.

## Supplementary Information

ESM 1 (DOCX 31 kb)

## Footnotes

1 We have also analyzed the data using the classic RM-ANOVAs. The results were mostly consistent with the findings of (G)LMMs except Experiment 2. See detailed report in the supplementary materials.

2 However, this interaction between interval and target location on ACC was significant in the RM-ANOVA analysis, *F*(2, 110) = 3.621, *p* = .030, η_p_^2^ = .062, which reveals the prioritization of only the temporally congruent high-long location at the long interval and the prioritization of both high-probability locations at the short interval compared with low-probability locations. See details in the supplementary materials.

3 All the awareness related control factors were not included in the GLMMs investigating the three-way interaction because of model convergence.

## References

[CR1] Awh E, Belopolsky AV, Theeuwes J (2012). Top-down versus bottom-up attentional control: A failed theoretical dichotomy. Trends in Cognitive Sciences.

[CR2] Barr DJ, Levy R, Scheepers C, Tily HJ (2013). Random effects structure for confirmatory hypothesis testing: Keep it maximal. Journal of Memory and Language.

[CR3] Bates D, Mächler M, Bolker B, Walker S (2015). Fitting linear mixed-effects models using lme4. Journal of Statistical Software.

[CR4] Boettcher SEP, Shalev N, Wolfe JM, Nobre AC (2022). Right place, right time: Spatiotemporal predictions guide attention in dynamic visual search. Journal of Experimental Psychology. General.

[CR5] Campbell JID, Thompson VA (2012). MorePower 6.0 for ANOVA with relational confidence intervals and Bayesian analysis. Behavior Research Methods.

[CR6] Cave K, Bush WS, Taylor TGG (2010). Split attention as part of a flexible attentional system for complex scenes: Comment on Jans, Peters, and De Weerd (2010). Psychological Review.

[CR7] Chun MM, Jiang Y (1998). Contextual cueing: Implicit learning and memory of visual context guides spatial attention. Cognitive Psychology.

[CR8] Chun MM, Jiang Y (1999). Top-down attentional guidance based on implicit learning of visual covariation. Psychological Science.

[CR9] Coull JT, Nobre AC (1998). Where and when to pay attention: The neural systems for directing attention to spatial locations and to time intervals as revealed by both PET and fMRI. Journal of Neuroscience.

[CR10] Druker M, Anderson B (2010). Spatial probability aids visual stimulus discrimination. Frontiers in Human Neuroscience.

[CR11] Failing M, Feldmann-Wüstefeld T, Wang B, Olivers C, Theeuwes J (2019). Statistical regularities induce spatial as well as feature-specific suppression. Journal of Experimental Psychology: Human Perception and Performance.

[CR12] Failing M, Theeuwes J (2018). Selection history: How reward modulates selectivity of visual attention. Psychonomic Bulletin & Review.

[CR13] Fiser J, Aslin RN (2001). Unsupervised statistical learning of higher-order spatial structures from visual scenes. Psychological Science.

[CR14] Gabbay C, Zivony A, Lamy D (2019). Splitting the attentional spotlight? Evidence from attentional capture by successive events. Visual Cognition.

[CR15] Geng JJ, Behrmann M (2002). Probability cuing of target location facilitates visual search implicitly in normal participants and patients with hemispatial neglect. Psychological Science.

[CR16] Geng JJ, Behrmann M (2005). Spatial probability as an attentional cue in visual search. Perception & Psychophysics.

[CR17] Hackley SA, Valle-Inclán F (1998). Automatic alerting does not speed late motoric processes in a reaction-time task. Nature.

[CR18] Huang C, Vilotijević A, Theeuwes J, Donk M (2021). Proactive distractor suppression elicited by statistical regularities in visual search. Psychonomic Bulletin & Review.

[CR19] Jaeger TF (2008). Categorical data analysis: Away from ANOVAs (transformation or not) and towards logit mixed models. Journal of Memory and Language.

[CR20] Jans B, Peters JC, De Weerd P (2010). Visual spatial attention to multiple locations at once: The jury is still out. Psychological Review.

[CR21] Jiang YV, Swallow KM, Rosenbaum GM, Herzig C (2013). Rapid acquisition but slow extinction of an attentional bias in space. Journal of Experimental Psychology: Human Perception and Performance.

[CR22] Klein RM (2000). Inhibition of return. Trends in Cognitive Sciences.

[CR23] Li A-S, Theeuwes J (2020). Statistical regularities across trials bias attentional selection. Journal of Experimental Psychology: Human Perception and Performance.

[CR24] Logan GD (1996). The CODE theory of visual attention: An integration of space-based and object-based attention. Psychological Review.

[CR25] Los SA, Kruijne W, Meeter M (2014). Outlines of a multiple trace theory of temporal preparation. Frontiers in Psychology.

[CR26] Los SA, Kruijne W, Meeter M (2017). Hazard versus history: Temporal preparation is driven by past experience. Journal of Experimental Psychology: Human Perception and Performance.

[CR27] Los SA, Nieuwenstein J, Bouharab A, Stephens DJ, Meeter M, Kruijne W (2021). The warning stimulus as retrieval cue: The role of associative memory in temporal preparation. Cognitive Psychology.

[CR28] Maljkovic V, Nakayama K (1994). Priming of pop-out: I. Role of features. Memory & Cognition.

[CR29] Mathôt S, Schreij D, Theeuwes J (2012). OpenSesame: An open-source, graphical experiment builder for the social sciences. Behavior Research Methods.

[CR30] Mattiesing RM, Kruijne W, Meeter M, Los SA (2017). Timing a week later: The role of long-term memory in temporal preparation. Psychonomic Bulletin & Review.

[CR31] McMains SA, Somers DC (2004). Multiple spotlights of attentional selection in human visual cortex. Neuron.

[CR32] Miniussi C, Wilding EL, Coull JT, Nobre AC (1999). Orienting attention in time: Modulation of brain potentials. *Brain: A*. Journal of Neurology.

[CR33] Näätänen R (1971). Non-aging fore-periods and simple reaction time. Acta Psychologica.

[CR34] Niemi P, Näätänen R (1981). Foreperiod and reaction time. Psychological Bulletin.

[CR35] Nobre, A. C., Correa, A., & Coull, J. T. (2007). The hazards of time. *Current Opinion in Neurobiology, 17*(4), 465–470. 10.1016/j.conb.2007.07.006 10.1016/j.conb.2007.07.006 17709239

[CR36] Nobre AC, van Ede F (2018). Anticipated moments: Temporal structure in attention. Nature Reviews Neuroscience.

[CR37] Olson IR, Chun MM (2001). Temporal contextual cuing of visual attention. Journal of Experimental Psychology: Learning, Memory, and Cognition.

[CR38] Pfeuffer CU, Aufschnaiter S, Thomaschke R, Kiesel A (2020). Only time will tell the future: Anticipatory saccades reveal the temporal dynamics of time-based location and task expectancy. Journal of Experimental Psychology: Human Perception and Performance.

[CR39] Posner M, Snyder C, Davidson B (1980). Attention and the detection of signals. Journal of Experimental Psychology: General.

[CR40] Rieth CA, Huber DE (2013). Implicit learning of spatiotemporal contingencies in spatial cueing. Journal of Experimental Psychology: Human Perception and Performance.

[CR41] Rolke B, Ulrich R, Nobre AC, Coull JT (2010). On the locus of temporal preparation: Enhancement of pre-motor processes. *Attention and time*.

[CR42] RStudio Team. (2021). RStudio: Integrated Development Environment for R. http://www.rstudio.com/

[CR43] Saffran JR, Aslin RN, Newport EL (1996). Statistical learning by 8-month-old infants. Science.

[CR44] Salet, J. M., Kruijne, W., van Rijn, H., Los, S. A., & Meeter, M. (2022). FMTP: A unifying computational framework of temporal preparation across time scales. *Psychological Review. Advance online publication.*10.1037/rev0000356 10.1037/rev0000356 35420847

[CR45] Steinborn MB, Langner R (2012). Arousal modulates temporal preparation under increased time uncertainty: Evidence from higher-order sequential foreperiod effects. Acta Psychologica.

[CR46] Theeuwes J (1991). Cross-dimensional perceptual selectivity. Perception & Psychophysics.

[CR47] Theeuwes J (1992). Perceptual selectivity for color and form. Perception & Psychophysics.

[CR48] Theeuwes J (2019). Goal-driven, stimulus-driven, and history-driven selection. Current Opinion in Psychology.

[CR49] Thomaschke R, Dreisbach G (2013). Temporal predictability facilitates action, not perception. Psychological Science.

[CR50] Thomaschke R, Dreisbach G (2015). The time-event correlation effect is due to temporal expectancy, not to partial transition costs. Journal of Experimental Psychology: Human Perception and Performance.

[CR51] Thomaschke R, Kiesel A, Hoffmann J (2011). Response specific temporal expectancy: Evidence from a variable foreperiod paradigm. Attention, Perception, & Psychophysics.

[CR52] Trillenberg P, Verleger R, Wascher E, Wauschkuhn B, Wessel K (2000). CNV and temporal uncertainty with 'ageing' and 'nonageing' S1-S2 intervals. Clinical Neurophysiology.

[CR53] Turk-Browne NB, Jungé J, Scholl BJ (2005). The automaticity of visual statistical learning. Journal of Experimental Psychology. General.

[CR54] van Moorselaar D, Daneshtalab N, Slagter HA (2021). Neural mechanisms underlying distractor inhibition on the basis of feature and/or spatial expectations. Cortex.

[CR55] Vangkilde S, Petersen A, Bundesen C (2013). Temporal expectancy in the context of a theory of visual attention. Philosophical Transactions of the Royal Society B: Biological Sciences.

[CR56] Visalli A, Capizzi M, Ambrosini E, Kopp B, Vallesi A (2021). Electroencephalographic correlates of temporal Bayesian belief updating and surprise. NeuroImage.

[CR57] Visalli A, Capizzi M, Ambrosini E, Mazzonetto I, Vallesi A (2019). Bayesian modeling of temporal expectations in the human brain. NeuroImage.

[CR58] Volberg G, Thomaschke R (2017). Time-based expectations entail preparatory motor activity. Cortex.

[CR59] Wagener A, Hoffmann J (2010). Temporal cueing of target-identity and target-location. Experimental Psychology.

[CR60] Wang B, Theeuwes J (2018). Statistical regularities modulate attentional capture. Journal of Experimental Psychology: Human Perception and Performance.

[CR61] Wang B, Theeuwes J (2018). How to inhibit a distractor location? Statistical learning versus active, top-down suppression. Attention, Perception, & Psychophysics.

[CR62] Wang B, Theeuwes J (2018). Statistical regularities modulate attentional capture independent of search strategy. Attention, Perception, & Psychophysics.

[CR63] Wang B, van Driel J, Ort E, Theeuwes J (2019). Anticipatory distractor suppression elicited by statistical regularities in visual search. Journal of Cognitive Neuroscience.

[CR64] Wang L, Wang B, Theeuwes J (2021). Across-trial spatial suppression in visual search. Attention, Perception, & Psychophysics.

[CR65] Xu Z, Los SA, Theeuwes J (2021). Attentional suppression in time and space. Journal of Experimental Psychology. Human Perception and Performance.

[CR66] Zhao J, Al-Aidroos N, Turk-Browne NB (2013). Attention is spontaneously biased toward regularities. Psychological Science.

