# Supplementary material for: Statistical learning of spatiotemporal regularities dynamically guides visual attention across space
Source: Atten Percept Psychophys. 2022 Oct 7;85(4):1054–72. doi: 10.3758/s13414-022-02573-5 (PMC10167174; doi:10.3758/s13414-022-02573-5)
Supplement: Supplementary file 1 — (DOCX 31 kb) [file 13414_2022_2573_MOESM1_ESM.docx]

**Supplementary Materials**

**Statistical learning of spatiotemporal regularities dynamically guides visual attention across space**

Zhenzhen Xu, Jan Theeuwes & Sander A. Los

In addition to the (G)LMM analyses reported in the main text, we also analyzed the data by using the classic repeated measures ANOVAs (RM-ANOVAs). The filtered data were averaged for each participant and each condition, and then entered in a RM-ANOVA with interval (500 ms, 1500 ms) and target location (high-short, high-long, low) as repeated factors. The Greenhouse–Geisser correction was applied whenever there was evidence for a violation of the sphericity assumption based on Mauchly’s test. Least significance difference (LSD) correction was used in the post hoc tests.

**Results**

**Experiment 1**

The RM-ANOVA on RT revealed a significant main effect of interval, *F*(1, 40) = 7.729, *p* = .008, η_p_^2^ = .162. There was also a significant main effect of target location, *F*(1.480, 59.202) = 28.674, *p* < .001, η_p_^2^ = .418. Compared to the mean RT for the low probability target locations, the performance for the high-short location and the high-long location were both significantly faster (*ps* ≤ .006). There was also a significant difference between the high-short location and the high-long location (*p* < .001). Importantly, the interaction between interval and target location was significant, *F*(1.721, 68.854) = 4.341, *p* = .021, η_p_^2^ = .098. The corresponding RM-ANOVA on ACC only revealed a significant main effect of target location, *F*(2, 80) = 15.815, *p* < .001, η_p_^2^ = .283. Participants responded more accurately when the target appeared at the high-short location than at either the high-long location (*p* < .001) or any of the low probability locations. There was no significant difference between the high-long location and the low probability locations (*p* = .137). No other effect was significant (*F*s <1).

Post hoc tests were conducted to further investigate the significant interaction between interval and target location on the RT data. Across trials with the 500-ms interval, mean RT was significantly faster when the target appeared at the high-short location than when it appeared at any one of the low probability target locations, *t*(40) = 9.995, *p* < .001, *d* = 1.561. The mean RT for the high-short location was also significantly faster than that for the high-long location, *t*(40) = 4.603, *p* < .001, *d* = 0.719, indicating better performance for the temporally congruent location than the temporally incongruent location at the 500-ms interval. In contrast, there was no significant difference between the mean RTs for the temporally incongruent high-long location and the neutral low probability target locations, *t*(40) = 1.923, *p* = .062, *d* = 0.300.

Across trials with the 1500-ms interval, the mean RT for the high-long location was significantly shorter than that for the low probability locations, *t*(40) = 3.572, *p* < *.*001, *d* = 0.558, suggesting a behavioral advantage for the temporally congruent location than the neutral locations at the 1500-ms interval. Interestingly, the mean RT was also significantly faster when the target was presented at the temporally incongruent high-short location than when it was presented at any of the neutral low probability target locations, *t*(40) = 7.308, *p* < *.*001, *d* = 1.141. The mean RT for the high-short location was also significantly shorter than that for the high-long location at the 1500-ms interval, *t*(40) = 2.415, *p* = .020, *d* = 0.377.

Furthermore, post hoc tests showed that the reduction of RT from the 500-ms to the 1500-ms interval condition differed between target locations. For trials in which the target appeared at any of the neutral low probability locations, the mean RT was significantly shorter for the 1500-ms interval condition than the 500-ms interval condition, *t*(40) = 2.563, *p* = .014, *d* = 0.400, reflecting the effect of temporal preparation under a uniform interval distribution. When the target appeared at the high-long location, there was also a reduction of RT from the non-associated 500-ms interval condition to the associated 1500-ms interval condition, *t*(40) = 3.874, *p* < *.*001, *d* = 0.605. By contrast, there was no significant difference between the associated 500-ms interval condition and the non-associated 1500-ms interval condition when the target appeared at the high-short location, *t*(40) = 0.073, *p* = .942, *d* = 0.011.

**Experiment 2**

A RM-ANOVA on RT showed no significant main effect of interval, *F*(1, 55) = 0.033, *p* = .857, η_p_^2^= .001, revealing an approximately flat RT – interval function in all the locations. This indicates that the manipulation of interval distribution worked as expected. There was a significant main effect of target location, *F*(2, 110) = 40.527, *p* < .001, η_p_^2^ = .424. Compared to the mean RT for the low probability target locations, the performance for the high-short location and the high-long location were both significantly faster (*ps* < .001). There was no significant difference between the high-short location and the high-long location (*p* = .127). The interaction between interval and target location was also not significant, *F*(1.795, 98.716) = 1.381, *p* = .256, η_p_^2^ = .024. The analysis of ACC revealed no significant main effect of interval, *F*(1, 55) = 0.027, *p* = .870, η_p_^2^ = .000, but a significant main effect of target location, *F*(2, 110) = 11.063, *p* < .001, η_p_^2^ = .167. Participants responded more accurately for the two high probability locations than for the low probability locations (*ps* ≤ .001). More importantly, there was a significant interaction between interval and target location on ACC data, *F*(2, 110) = 3.621, *p* = .030, η_p_^2^ = .062.

Post hoc tests for the interaction effect on ACC showed that after the interval of 500 ms, the mean ACC for the temporally congruent high-short location was significantly higher than that for the neutral low probability target locations, *t*(55) = 5.169, *p* < .001, *d* = 0.691. Also, the mean ACC for the temporally incongruent high-long location was also significantly higher than that for the neutral low probability target locations, *t*(55) = 3.931, *p* < .001, *d* = 0.525. There was no significant difference between the high-short location and the high-long location, *t*(55) = 0.753, *p* = .455, *d* = 0.101, indicating the attentional bias towards both the high probability locations at the short interval. When the search display was presented after the interval of 1500 ms, there was no significant difference between the temporally incongruent high-short location and the low probability target locations, *t*(55) = 1.583, *p* = .119, *d* = 0.212. In contrast, the mean ACC for the temporally congruent high-long location was significantly higher than that for the low probability target locations, *t*(55) = 4.310, *p* < .001, *d* = 0.576. The mean ACC for the high-short location and the high-long location was not significantly different, *t*(55) = 1.461, *p* = .150, *d* = 0.195. Thus, we observed the prioritization of both two high probability locations compared to the low probability location at the short interval and only the prioritization of the temporally congruent high-long location compared to the low probability location at the long interval. Post hoc tests revealed no significant effect of interval on ACC for any one of the target locations (*ps* > .07), consistent with earlier findings that temporal preparation is constant over time under the exponential distribution.

**Experiment 3**

A RM-ANOVA on RT showed a significant main effect of interval, *F*(1, 58) = 20.580, *p* < .001, η_p_^2^ = .262. There was also a significant main effect of target location, *F*(1.613, 93.532) = 47.750, *p* < .001, η_p_^2^ = .452. Compared to the mean RT for the low probability target locations, the performance for the high-short location and the high-long location were both significantly faster (*ps* < .001). There was no significant difference between the high-short location and the high-long location (*p* = .214). Importantly, the interaction between interval and target location was significant, *F*(2, 116) = 3.711, *p* = .027, η_p_^2^ = .060. The corresponding RM-ANOVA on ACC only revealed a significant main effect of target location, *F*(1.696, 98.392) = 14.810, *p* < .001, η_p_^2^ = .203. Participants were less accurate when the target appeared at any one of the low probability target locations than when it appeared at the high-short location (*p* < .001) or the high-long location (*p* < .001). There was no significant difference between the mean ACCs for the high-short location and the high-long location (*p* = .885). There were no other significant results (*F*s < 1).

Post hoc tests for the significant interaction effect on RT showed that, after the interval of 500 ms, participants responded significantly slower when the target appeared at any of the neutral low probability target locations than when it appeared at the temporally congruent high-short location, *t*(58) = 11.174, *p* < .001, *d* = 1.455. However, the RT for the high-long location was also significantly faster than that for the low probability locations, *t*(58) = 4.770, *p* < .001, *d* = 0.621. The difference between the mean RTs for the high-short location and the high-long location approached significance, *t*(58) = 1.977, *p* = .053, *d* = 0.257, showing a slightly shorter mean RT for the temporally congruent high probability location than the temporally incongruent high probability location at the short interval. After the interval of 1500 ms, the mean RT for the low probability target locations was significantly longer than that for the high-long location, *t*(58) = 8.006, *p* < .001, *d* = 1.042, and for the high-short location, *t*(58) = 10.560, *p* < .001, *d* = 1.375. The mean RTs for the high-long location and the high-short location were not significantly different, *t*(58) = 0.116, *p* = .908, *d* = 0.015. In addition, post hoc tests revealed that there was a significant difference between the two intervals for all the target locations (*ps* < .04), showing a shorter RT for the 1500-ms interval condition compared to the 500-ms interval condition at these locations.

Overall, the results of classic ANOVA were in accordance with the findings from the (G)LMM except the significant interaction of target location and interval on ACC in Experiment 2.
